# Pain in Chronic Pancreatitis During the COVID-19 Lockdown: Has It Given Us a New Dimension for Treatment?

**DOI:** 10.7759/cureus.13423

**Published:** 2021-02-18

**Authors:** Kunal Parasar, Shantam Mohan, Aaron G John, Utpal Anand

**Affiliations:** 1 Surgical Gastroenterology, All India Institute of Medical Sciences, Patna, IND; 2 Gastroenterology, All India Institute of Medical Sciences, Patna, IND

**Keywords:** chronic pancreatitis, depression, covid-19, lockdown, psycho-social

## Abstract

Background

Prolonged lockdown in our country provided us with a unique opportunity to study the interplay of psychosocial impact on pain in surgically treated patients of chronic pancreatitis.

Methods

Forty-one patients who underwent surgery for chronic pancreatitis in the last 24 months were followed up, of which 27 were enrolled. The data were collected telephonically. Pain was assessed using the numeric pain rating (NPR) scale and depression using Patient Health Questionnaire (PHQ) 9. In patients having recent onset pain during the lockdown, oral tramadol 50 mg and amitryptiline 25 mg were prescribed and reassessed after two weeks.

Results

Of the 25 pain-free patients in February (pre-lockdown), 14 developed pain of varying intensity during the lockdown and were prescribed medications. Twelve out of 14 patients had very good resolution of pain after two weeks of medication.

Conclusions

Operated patients with chronic pancreatitis who developed new-onset depression and pain responded well to low-dose anti-depressants in addition to analgesics. This study gives indirect, objective evidence that covert depression leading to pain in chronic pancreatitis is often downplayed and interpreted as poor results of surgery.

## Introduction

The current coronavirus disease (COVID-19) pandemic has influenced the natural course of various diseases unrelated to the coronavirus, with numerous factors affecting it. Several countries have implemented a lockdown of varying degrees, to decrease the spread of this deadly coronavirus. The prolonged lockdown in our country provided us with a unique opportunity to study the interplay of psychosocial impact on pain in surgically treated patients of chronic pancreatitis. Recurrent epigastric pain of varying intensity and frequency is the most common presentation of chronic pancreatitis and leads to poor quality of life (QOL) in these patients [1-2]. During initial studies, the etiopathogenesis of pain was thought to be somatic and treatment centered around analgesics, but the results were consistently poor. Further research moved from somatic pain theory to the neurobiological etiology of pain [3-4]. Based on the newer theory, many other groups of medicines and interventional therapies came to the forefront but the long-term results remain poor. Even though there are recommendations for using low-dose antidepressants in any form of chronic pain, including chronic pancreatitis, objective evidence is lacking [5]. There are a few studies already published on depression in chronic pancreatitis. However, this lockdown has given a uniform psychosocial environment for the entire group of patients with chronic pancreatitis who underwent surgical intervention at our center in the last 24 months.

## Materials and methods

We prospectively analyzed our retrospectively maintained database from April 2018 to March 2020 at the department of surgical gastroenterology, All India Institute of Medical Sciences, Patna. During this period, 88 patients with chronic pancreatitis were evaluated, out of which 41 patients underwent surgery (Frey's or lateral pancreaticojejunostomy) (Table 1).

**Table 1:** Demographic and Clinical Details of Patients

Characteristic	(n=31)
Mean age (range), years	30.25 (15 - 68)
Gender (Male/Female), n	22/9
Mean disease duration (range), months	41.5 (2 - 132)
Diarrhea, n	3 (9.67%)
Diabetes, n	8 (25.8%)
Surgical treatment	
Lateral pancreatojejunostomy	10
Frey's procedure	21

Out of 41 patients, 31 were under regular follow-up. The idea of this study was generated, as after two weeks of the implementation of lockdown, three patients operated within the last two years, who were asymptomatic before the lockdown presented to us with the complaint of pain, which was mild to moderate in intensity, dull aching, in the epigastrium and radiating to the back. We initially treated them with oral tramadol 50 mg, which was ineffective. On taking a detailed history, we were able to elicit evidence of recent onset depression due to the ongoing lockdown. So we added oral amitryptiline 25 mg and found good results in all three patients.

This led us to plan a study, which we conducted by calling up all operated patients who have been in regular follow-up at our center and asking them about their pain status in February (pre-lockdown), in the first week of May (during the lockdown period), and then again after two weeks of medication while still being in lockdown. The pain was measured with 11 points numeric pain rating (NPR) scale. This NPR scale has been validated for various chronic pain syndromes [6]. Assessment of depression was done by PHQ 9 (Table 2) [7-8].

**Table 2:** PHQ-9 Patient Depression Questionnaire Responses PHQ-Patient Health Questionnaire

	(n=27)
Depression as assessed with PHQ-9	
Minimal (0-4)	1
Mild (5-9)	7
Moderate (10-14)	17
Moderately severe (15-19)	2
Severe (20-27)	-
Mean PHQ-9 score (range)	11 (3 – 18)

Ethical clearance was obtained. We tried calling up all 31 patients under routine follow-up but were able to contact only 27 patients.

## Results

**Figure 1:**
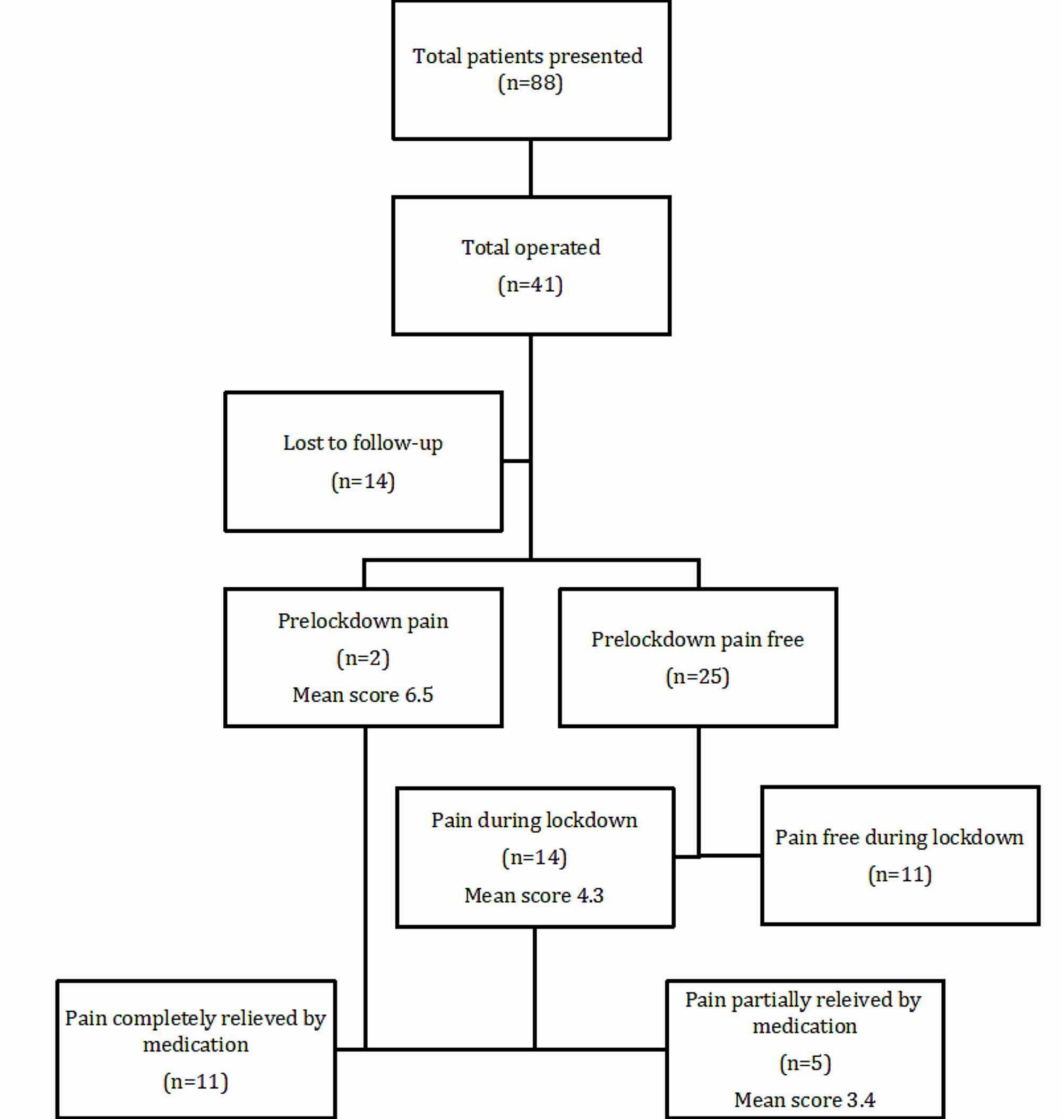
CONSORT flow diagram CONSORT: Consolidated Standards of Reporting Trials

Out of the 27 patients studied, two had pain in the month of February and before the lockdown occurred, but the intensity had increased in the month of May (Figure 1).

The rest of the 25 patients were pain-free in February (Table 3).

**Table 3:** Characteristics of Pain as Measured by the NPR Scale NPR: Numeric Pain Rating

	(n=27)
Patients with pre-lockdown pain	2
Patients with pain during the lockdown	16
Mean pain score during the lockdown period (range)	4.5(3 - 8)
Pain completely relieved by medication	11
Pain partially relieved by medication	5
Mean pain score in the patients with pain (range)	3.4 (1 - 5)

Out of the remaining 25 pain-free patients, 14 developed pain of varying intensity in the month of May. Even though the intensity of pain was variable, the nature of pain was similar in all 14 patients. We prescribed oral tramadol with amitriptyline in all 14 patients. Twelve out of 14 patients had very good resolution of pain after two weeks of medication. Three patients out of 27 had preoperative exocrine insufficiency and were on postoperative enzyme supplementation. None of the other 24 patients had any deterioration of exocrine insufficiency based on symptomatology. Getting data on endocrine insufficiency proved difficult, as most of them couldn't get their blood sugar examined due to the lockdown.

## Discussion

Pain is the most common symptom for which patients seek treatment in chronic pancreatitis [1-2]. A ton of research has been done to elucidate the various mechanisms of pain so that therapeutic benefit could be given to patients either through medicines or through endoscopic and surgical intervention. Modern medicine has been able to alleviate pain significantly with the above-said measures. But long-term pain-free improved quality of life (QOL) is still a distant dream.

Traditionally, pancreatic ductal and parenchymal hypertension, as well as pancreatic morphological changes, were considered to be the therapeutic challenges that were dealt with by endoscopic and surgical intervention [9-13]. But the long-term results were not as expected, which forced physicians to look beyond the morphological changes. Central cortical and spinal sensitization, impaired inhibitory pain modulation, altered pancreatic nociception along with pancreatic neuropathy and neuroplasticity, increased cholecystokinin, norepinephrine, and diabetic neuropathy have all been studied as other causes of pain [14-21]. Therapeutic interventions directed to all these mechanisms of pain are being tried. But the results as of now are not very encouraging [3-4].

Of all the mechanisms, depression leading to pain in chronic pancreatitis has not been studied well [22-23]. There is a very complex interplay between patient psychology and pain. The COVID-19 pandemic led to a prolonged lockdown in India, which was an unforeseen circumstance. This lockdown has allowed us to indirectly study the role of psychosomatic interplay in chronic pancreatitis pain. COVID-19, apart from its evident physical effects in infected cases, has seriously affected public mental health due to the imposition of lockdown. Different studies have pointed out an evident increase in psychological distress, depression, anxiety, and stress [24-27]. Based on this evidence and our aforementioned experiences with our cases, we surmised that psychological distress indeed might have played a role in the increase in pain in these groups of patients.

Based on telephonic conversations, covert depression of various magnitudes was present in all the patients. A noteworthy point in our study was that all the patients were surgically treated for the disease and were having good results. Still, more than 50% of our patient population, who was pain-free before lockdown, complained of typical pancreatic pain after six weeks of lockdown and got excellent results with antidepressants combined with a non-opioid analgesic.

It may be inferred that chronic pain leads to poor quality of life in patients, which, in the long term, leads to depression. This depression further propagates the pain, making it a vicious cycle. The addition of low-dose antidepressants could have helped us to break this cycle giving excellent results. It is evident from this study that the correlation between depression and pain is not linear. This study gave us indirect evidence that the mental health of patients is one of the pain pathways of pain, which we end up neglecting the most. We need further research to study this complex interplay between psychosocial aspects and pain.

The strength of our study was a very simple observational study design with an opportunity provided by circumstances. The drawback of this study is its small sample size and the indirect inference of the result. But this small study gives us an insight into a problem that deserves more attention from modern medicine.

## Conclusions

Despite all the currently available therapeutic interventions, the long-term results of pain-free good quality of life is still not possible in chronic pancreatitis. One of the possible causes that has not been given due attention is covert depression associated with chronic pancreatitis. The addition of low-dose anti-depressants may help break this vicious cycle of chronic pain, poor QOL, and depression and provide a good pain-free life.
